# Myeloperoxidase as a biomarker in periodontal disease: electrochemical detection using printed screen graphene electrodes

**DOI:** 10.1007/s10266-024-01043-8

**Published:** 2025-02-15

**Authors:** María C. Valdivieso, Ludy Ortiz, John J. Castillo

**Affiliations:** Escuela de Química, Universidad Industrial de Santander, Bucaramanga-Santander, Colombia

**Keywords:** Myeloperoxidase, Periodontal disease, Screen-printed graphene electrodes, Cyclic voltammetry, Sensor

## Abstract

Periodontal disease is a common oral health issue marked by inflammation and the breakdown of tissues. Early detection of biomarkers associated with periodontal disease (PD) can significantly aid in timely diagnosis and intervention. Myeloperoxidase (MPO) is an enzyme abundantly present in neutrophils and has been associated in the pathogenesis of PD. Here, we present a novel approach for the electrochemical detection of MPO using printed screen graphene electrodes coupled with principal component analysis (PCA) for data analysis. We employed cyclic voltammetry to characterize the electrochemical behavior of MPO using potassium ferrocyanide and hydrogen peroxide. The process was controlled by species diffusion on the electrode surface using a scan rate spanning from 10 to 400 mVs^−1^. In addition, we investigated the detection of hydrogen peroxide, a substrate of MPO, as a method to indirectly asses MPO electroactivity, leveraging a redox potential of − 500 mV. Saliva samples were collected and analyzed using the developed electrochemical sensor, followed by principal component analysis to differentiate between healthy and diseased samples based on MPO levels. Our findings demonstrate the feasibility of using printed screen graphene electrodes for the sensitive and selective detection of MPO, offering a promising approach for early diagnosis and monitoring of periodontal disease. In conclusion, our results highlight the potential of MPO as a robust biomarker for periodontal disease and highlight the utility of electrochemical sensing coupled with PCA analysis for sensitive and specific detection in clinical settings.

## Introduction

Periodontal disease (PD), a common oral health issue, includes a range of inflammatory diseases that impact the supporting tissues of the teeth, such as the gums, periodontal ligament, and alveolar bone [1]. Within this spectrum, periodontitis represents a severe and irreversible form characterized by the destruction of the periodontal ligament and alveolar bone. In this study, the term “periodontal disease” was used interchangeably with “periodontitis” to focus on the advanced stages of the condition. To provide a standardized framework for evaluating periodontitis, the 2017 World Workshop on the Classification of Periodontal and Peri-Implant Diseases introduced a staging and grading system. Staging assesses the severity, extent, and complexity of the disease, while grading evaluates the risk of progression and potential influence of systemic health factors [2]. These classifications are essential for tailoring treatment and understanding disease progression.

The relationship between PD and cardiovascular diseases has been of particular interest in recent studies, as they share some high-risk factors such as smoking and diabetes [3]. In addition, both diseases involve processes of inflammation and oxidative stress, where macrophages and neutrophils participate in the transformation of stable coronary arterial plaques into unstable lesions. With its multifactorial etiology and complex pathogenesis, periodontal disease presents significant challenges in diagnosis and management. While traditional clinical indicators like pocket depth and clinical attachment level remain essential in periodontal assessment, the quest for reliable biomarkers capable of early detection, accurate monitoring, and prognostic evaluation has intensified [4, 5].

According to the 2022 global report on oral health by the World Health Organization, severe PD is identified as the second most prevalent oral health condition worldwide, following untreated cavities in permanent teeth, with an estimated 1 billion cases [6]. In Colombia, approximately 61.8% of the population exhibits periodontitis across varying stages, predominantly manifesting in the moderate phase, particularly impacting individuals aged 45 and older [3]. The primary contributor to these statistics is inadequate oral hygiene practices. Limited resources allocated to oral disease prevention and treatment programs, as well as a lack of knowledge about the impact of advanced periodontitis on people’s quality of life, are reasons that justify the prevalence of this disease in the country.

Among the plethora of biomolecules implicated in periodontal inflammation, myeloperoxidase (MPO), an enzyme predominantly found in neutrophil granules, has emerged as a promising candidate. MPO is an enzyme primarily found in neutrophil granules, a kind of immune cell critical for the body’s defense system [7, 8]. It plays a vital role in innate immunity, particularly in the process of phagocytosis, where neutrophils engulf and destroy pathogens. The main function of MPO is to catalyze the production of hypochlorous acid (HOCl) and other reactive oxidants by utilizing hydrogen peroxide (H_2_O_2_) and chloride ions (Cl-) as substrates.

**Fig. 1:**
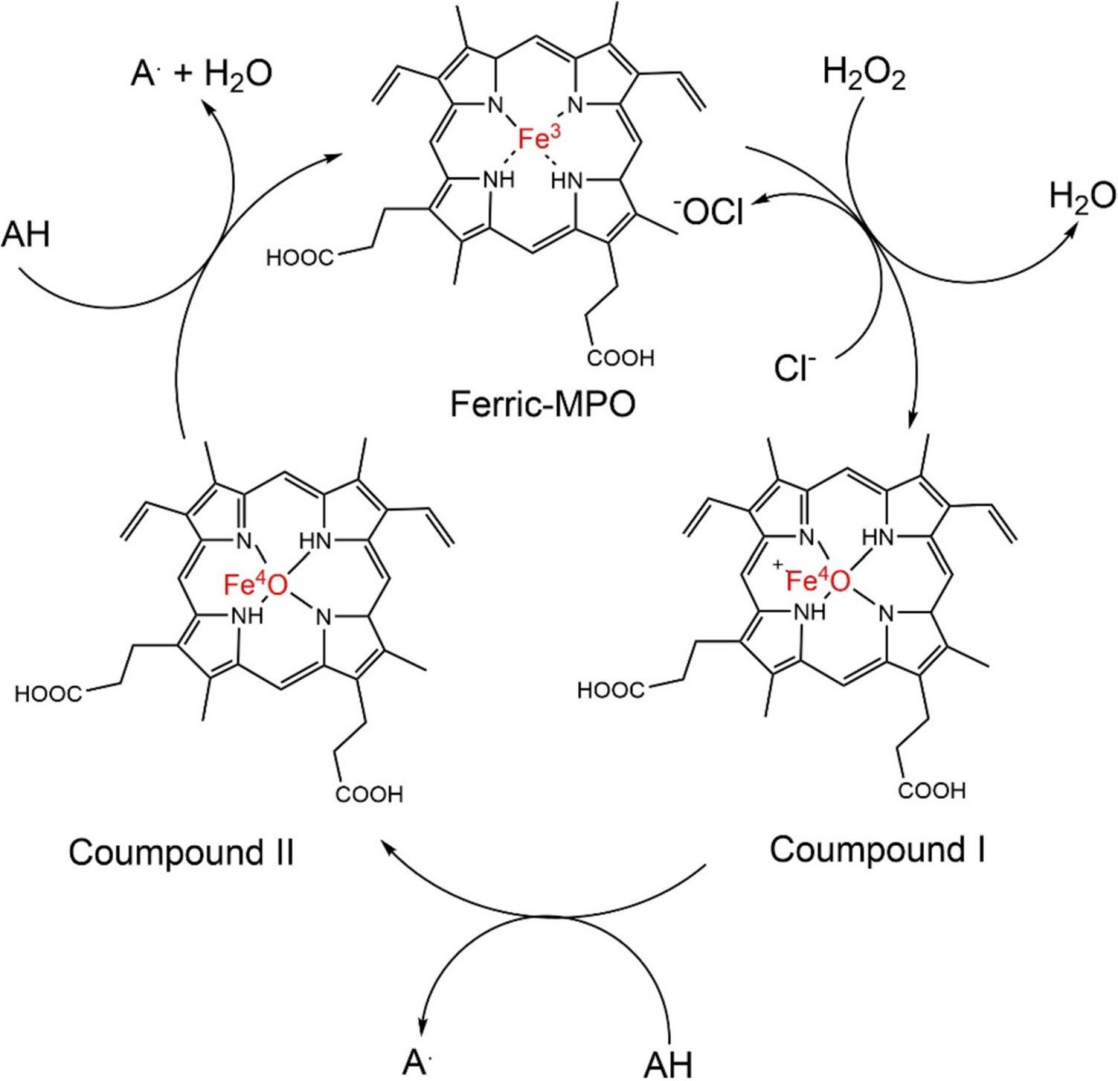
Catalytic cycle of MPO

Figure 1 shows the catalytic cycle of MPO. The cycle starts with the formation of compound I produced by the reaction of MPO with H_2_O_2._ In this state, MPO contains an oxoferryl group (Fe(IV) = O) and a porphyrin radical cation. Compound I act as a powerful oxidizing agent, capable of oxidizing a wide range of substrates, including proteins, lipids, and other molecules present in the phagolysosome. This oxidation process is crucial for killing pathogens. After oxidizing its substrate, Compound I is reduced back to its resting state, known as Compound II, through the transfer of electrons. Compound II still possesses some oxidizing ability but is less reactive than Compound I. Finally, Compound II is further reduced back to its original inactive state, completing the catalytic cycle of MPO. This regeneration typically involves the transfer of electrons from another substrate, such as ascorbate or thiocyanate, back to the enzyme.

The reactive oxidants, producing by MPO, are potent antimicrobial agents that help neutralize and destroy invading microorganisms, such as bacteria and fungi [9, 10]. Elevated MPO levels have been consistently associated with various inflammatory conditions, including periodontitis, reflecting the abundance and activity of neutrophils within the periodontal tissues [11]. Furthermore, MPO’s involvement in oxidative stress pathways and its ability to modulate host-microbial interactions underscore its potential as a biomarker for PD.

The conventional methods for MPO detection often entail labor-intensive procedures, limited sensitivity, and lengthy processing times, necessitating the exploration of innovative detection strategies to enhance diagnostic efficiency [12, 13]. In this context, the utilization of printed screen graphene electrodes (SPGE) presents a compelling avenue for electrochemical detection, offering advantages such as rapid response, high sensitivity, and ease of miniaturization [14–17]. Since its discovery in 2004, graphene has attracted significant attention as a cutting-edge nanomaterial due to its remarkable electronic and electrochemical characteristics, making it especially promising for the development of advanced biosensing technologies [18–21]. Increasing emphasis has been placed on augmenting graphene with functional molecules or alternative nanomaterials to facilitate its utilization across various domains, including biosensing, energy storage, and biofuel cells, among others. Enzymes [22], antibodies [23], metal nanoparticles [24], DNA [25], and quantum dots [26] represent a subset of the materials employed for the functionalization of graphene.

In 2022, Li et al., constructed an amperometry immunosensor for MPO detection by immobilizing anti-MPO on a modified glassy carbon electrode [27]. The sensor response was proportional to the concentration of MPO in the range of 5–300 ng/mL, with a detection limit of 0.2 ng/mL. In a similar study, Windmiller et al., developed an amperometry biosensor based on screen-printed strips for determining MPO levels [28]. This biosensor utilized 3,3',5,5'-tetramethylbenzidine (TMB) as a redox mediator to enable the quantification of MPO levels. The biosensor exhibited a liner range spanning from 3 to 18 U/L in both acetate buffer (pH 4.5) and human serum. Wen et al., developed an amperometry immunosensor for quantitative MPO detection in serum using trimetallic CuPdPt nanowire networks [8]. They successfully determined MPO levels in clinical serum samples, achieving a linear response within the MPO concentration range of 100 fg/mL–50 ng/mL and a low detection limit of 33 fg/mL. Finally, the most recent research, published in 2023 by Tao et al., involved the development of a flexible amperometry immunosensor for MPO detection using an electrode modified with quantum dots [29]. This sensor exhibited high sensitivity (31.6 fg/mL) and a wide detection range (1 pg/mL–1 ng/mL).

This study aims to evaluate the presence of MPO in saliva samples by the bioelectrochemical reduction of H_2_O_2_ using SPGE. A simple drop-casting procedure allows us to deposited saliva samples on the surface of the SPGE and measure the reduction current of H_2_O_2_ by cyclic voltammetry technique (CV). The obtained CVs coupled to PCA analysis allowed to develop an electrochemical method to detect the presence of MPO as a biomarker for PD in saliva samples.

## Materials and methods

The enzymatic activity of MPO (commercially acquired from Sigma Aldrich, USA) was determined using a colorimetric assay employing following the absorbance change occurring at 470 nm, when guaiacol is oxidized, a reaction catalyzed by MPO in the presence of H_2_O_2_, forming the amber-colored tetraguaiacol product, responsible for the absorption signal at 470 nm [17].

Electroanalytical measurements were performed with an Autolab PGSTAT101 device (Echo Chemie, Utrecht, the Netherlands) run by the NOVA 1.10.1.9 software (Metrohm, Filderstadt, Germany). Screen-printed graphene electrodes (L33 X W10 X H0.5 mm) modified with graphene (SPGE, 110GPH) were obtained from DropSens (Oviedo, Spain). These screen-printed electrodes (consisting of a working electrode of 4 mm in diameter, a silver rod pseudo-reference electrode, and an auxiliary carbon electrode) are disposable and are modified with graphene. CV experiments were carried out at 27 °C in different solutions. Before each CV experiment, the solutions were freed of oxygen bubbles through degasification with N_2_ and magnetic stirring for 30 s.

Prior to usage, bare electrodes were cleaned with ethanol and water, followed by thorough drying under a stream of nitrogen. Subsequently, 5 μL of MPO solution was applied onto the surface of screen-printed electrodes and air-dried for approximately 10 h. Following a 4-h incubation at 4 °C, the modified electrodes were gently rinsed with 10 mM phosphate buffer at pH 7.0 to remove any unbound MPO, and ensure the stable binding of the MPO enzyme to the electrode surface, facilitating the immobilization of MPO. Then CVs were running in the presence of H_2_O_2_ at different concentrations.

Saliva samples were obtained from the dental center of the Carlos Ardila Lulle clinic. 30 saliva samples (The sample size of 30 was determined as a convenience sample based on the availability of patients at the dental center during the study period) were collected from patients diagnosed with periodontitis, 10 from patients with gingivitis, and 5 from individuals with clinically healthy periodontal tissues (previously diagnosed by a basic periodontal test) who had previously signed a consent form allowing the analysis of these samples and informing them of potential risk factor diseases. Saliva samples were collected in the mornings, stored in 15 mL Falcon tubes at 4ºC, and analyzed within 24 h of collection. Prior to analysis, the samples were centrifuged at 12,000 g for 20 min. Then 16uL of saliva supernatant were deposited and allowed to adsorb onto each electrode for 2–4 h. After the sample was dried, 20uL of a 0.5 mM solution of H_2_O_2_ dissolved in 100 mM PBS buffer was added to the electrode surface.

CV measurements were used to study the electrocatalytic activity of MPO toward the reduction of H_2_O_2_ and to detect the presence of MPO in the saliva samples. CVs were carried out at a scan rate of 150 mV/s.

## Results

### Enzymatic activity of MPO

The enzymatic activity of the commercial MPO was assessed to evaluate its oxidation toward guaiacol yielding a value of 48.4 U/mL in a good concordance with other studies [17, 30]. The MPO was kept at 4 °C prior to the electrochemical experiments.

### Effect of the scan rate on the bioelectrocatalytic reduction of H_2_O_2_ by commercial MPO

**Fig. 2:**
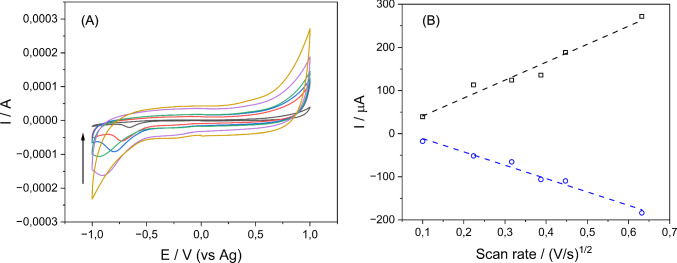
Cyclic voltammograms (**A**) and scan rate root vs cathodic and anodic currents (**B**) of MPO-SPGE with H_2_O_2_ 0.05 mM in 100 mM PBS pH 7.2 at different scan rates (10, 50, 100, 150, 200, and 400 mV/s)

CV experiments showed a linear relationship between the square root of the scan rate and the peak current, confirming a surface-controlled quasi-reversible process. Figure 2A displays CVs and the dependence of cathodic and anodic currents on the scan rate, supporting a diffusion-controlled mechanism [33].

The scan rate controls the rate of change in the applied potential on the electrode [31–33]. A high rate leads to a reduction in the diffusion layer thickness on the electrode, resulting in higher currents (Fig. 2B). However, for the reaction to be electrochemically reversible, it must occur in a diffusion-controlled environment of the redox species. This is evidenced by observing a linear relationship between the current peaks and the square root of the scan rate.

### Optimal concentration of bioelectrocatalytic reduction of H_2_O_2_ by the MPO

**Fig. 3:**
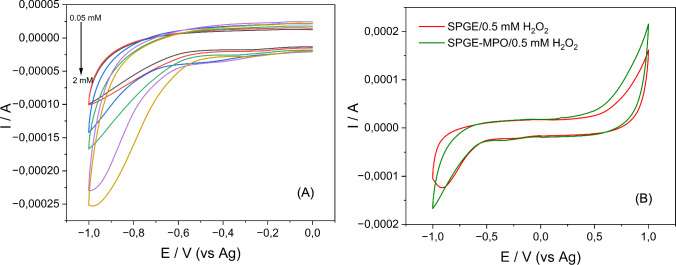
**A** CVs of MPO-SPGE at different concentrations of H_2_O_2_ (0.05, 0.1, 0.25, 0.5, 1, and 2 mM), scan rate 0.15 mV/s, **B** CVs of modified (green) and unmodified SPGE in the presence of 0.5 mM

A proper substrate choice and the concentration of H_2_O_2_ are crucial for achieving optimal enzyme performance. A highly concentrated solution can trigger enzyme inhibition due to excess substrate, slowing down the catalyzed reaction and hindering the transfer of charge generated by the enzyme–substrate interaction. This could result in information bias due to a decrease in the reported current. Different concentrations of H_2_O_2_ (0.05–2 mM) were tested. The results demonstrated a direct correlation between peroxide concentration and electrochemical response, with 0.5 mM identified as optimal based on CV symmetry (Fig. 3A). Control experiments showed a significant current increase upon MPO modification, indicating its catalytic role (Fig. 3B).

Figure 3A presents the effect of hydrogen peroxide concentrations detected by myeloperoxidase (MPO) deposited on the surface of SPGE measured by CV.

### Electrochemical detection of MPO in saliva samples

**Fig. 4:**
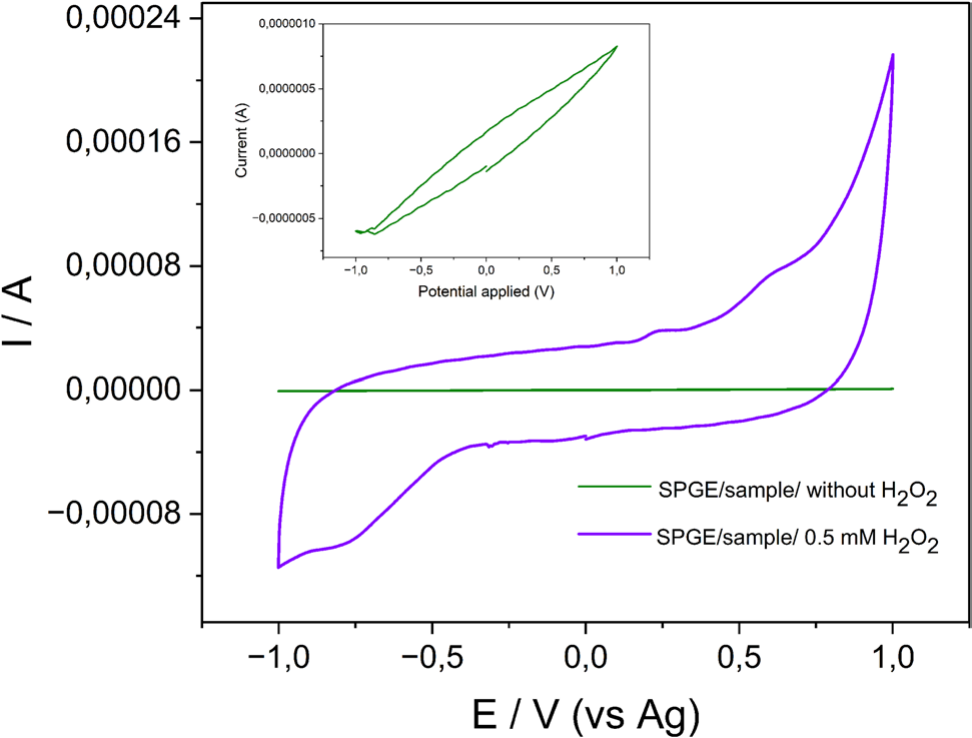
CVs of SPGE modified with saliva sample in the presence (purple) and absence (green) of H_2_O_2_. Inset: detail of CV of SPGE without H_2_O_2_. Scan rate 0.15 mV/s

**Fig. 5:**
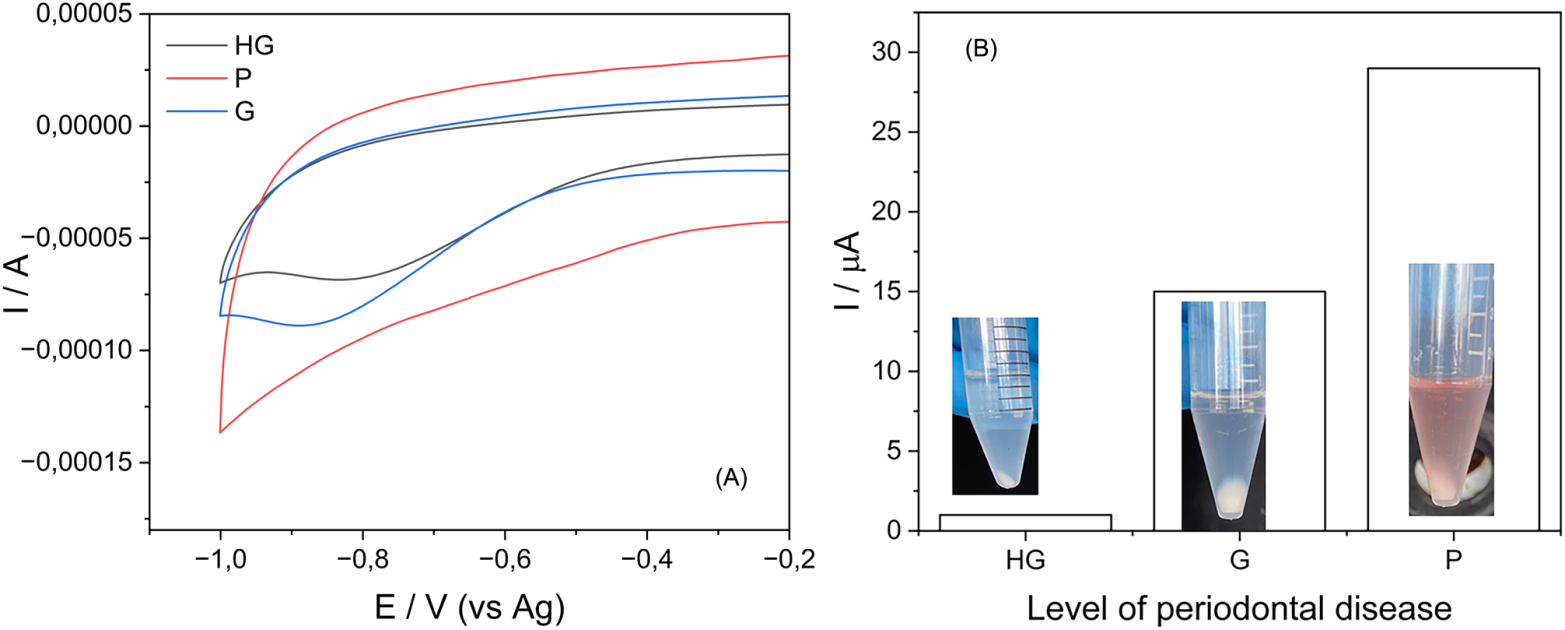
**A** CVs for MPO present in 3 saliva samples; **B** intensity of the current for bioelectrocatalytic reduction of H_2_O_2_ by MPO present in 3 saliva samples at three different levels of PD (*HG* healthy gingiva, no periodontal disease; *G* gingivitis and *P* periodontitis)

Before to start with the electrochemical study of the 37 saliva samples, a preliminary experiment was conducted to test the efficiency of the SPGE, measuring MPO levels in an unclassified saliva sample, i.e., without diagnosis based on PBT. CV analysis of unclassified saliva samples confirmed the ability of the SPGE to detect MPO activity in the presence of H_2_O_2_ (Fig. 4). For classified samples, patients with higher PD levels exhibited increased cathodic currents, correlating with MPO concentrations (Fig. 5).

As is shown in Fig. 4, the SPGE modified with the saliva sample exhibited a significantly current signal toward the reduction of H_2_O_2_. Conversely, the SPGE modified with saliva sample but lacking H_2_O_2_ displayed a weak cyclic CV with no discernible electroactive species, indicating an absence of signal current (see inset of Fig. 4).

Now we proceed to analyze 7 samples collected randomly in the Industrial University of Santander, and 30 samples collected from patients at the dental office of the Carlos Ardila Lulle Clinic. The samples taken on the campus were labeled as ‘unclassified’ since the participants were not aware of their periodontal health status. The samples taken at the dental office were labeled according to the presence or absence of PD, according to a basic periodontal test (BPT) [10, 34], considering the periodontal status recorded by medical staff in the informed consent.

Figure 5A shows the reduction region of H_2_O_2_ by MPO present in 3 saliva sample from different patients with healthy gingiva, gingivitis, and periodontitis, respectively. As expected, a higher current signal in patients presenting PD implies the presence of a higher MPO concentration, suggesting a greater reaction rate and consequently a higher current. On the other hand, Fig. 5B shows the values of the cathodic current for the saliva samples with different presence of MPO. These results are in a good agreement with the previous classification performed by the BPT.

### Principal component analysis of saliva samples

Principal component analysis (PCA) is a statistical method used to reduce the dimensionality of data and aid in its visualization [35]. It simplifies the complexity in high-dimensional data while retaining trends and patterns [36]. PCA could be useful as a statistical tool in the context of electrochemical methods aimed at detecting MPO as a biomarker in saliva samples. Given the intricate nature of electrochemical data, characterized by multiple variables and potential interdependencies, PCA offers a systematic approach to extract meaningful information and reduce dimensionality [37]. By condensing the complex electrochemical signals into principal components, PCA facilitates the identification of patterns and correlations within the dataset, enhancing the interpretability of results.

For this purpose and using the current values obtained, a PCA was conducted to segregate the groups according to the patients’ condition and determine if the current response was a useful parameter for this grouping. The PCA analysis allowed for the distribution of the samples according to their presence (red and purple color) or absence (green color) of periodontal disease.

**Fig. 6:**
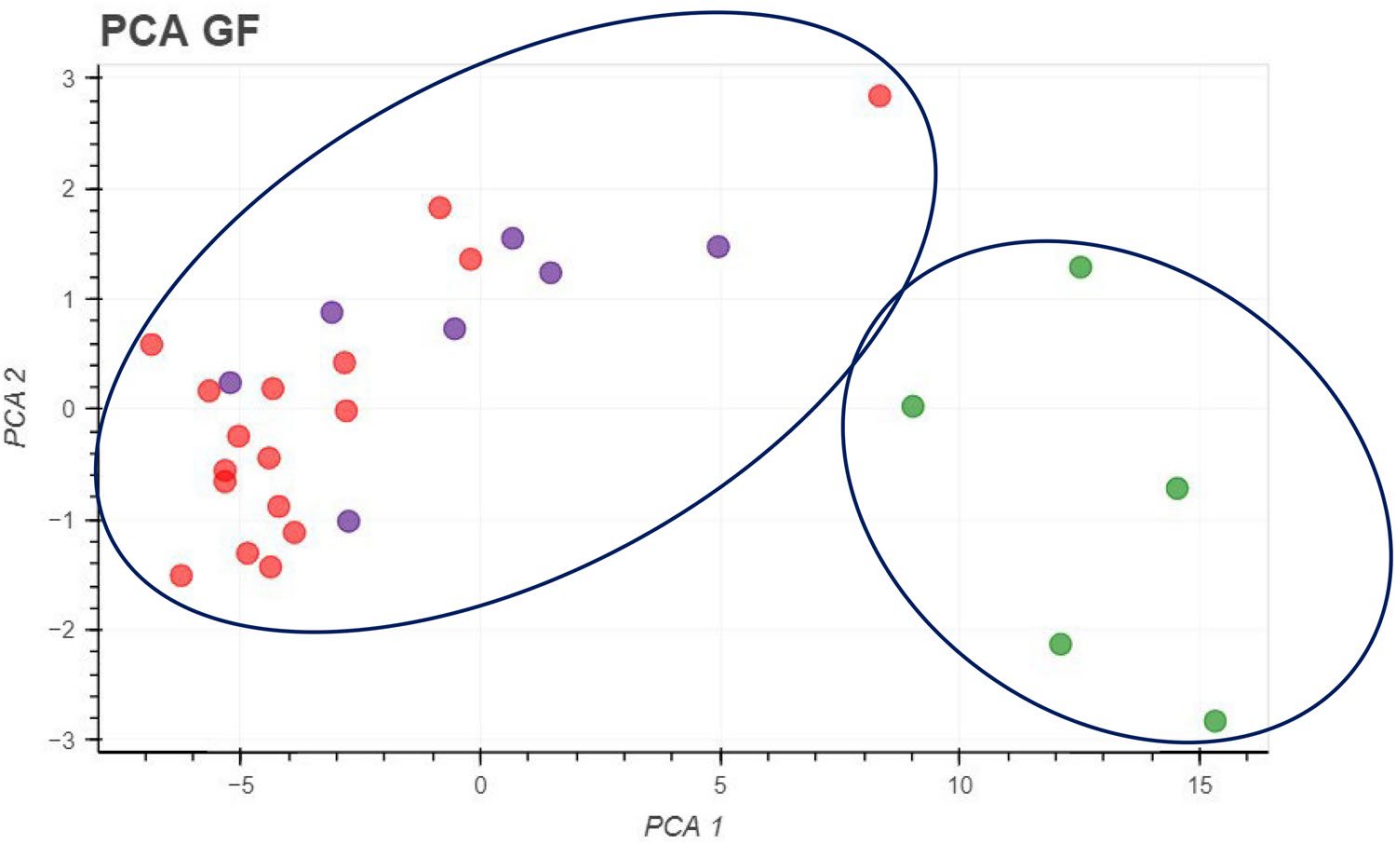
PCA of saliva samples using SPGE

Figure 6 shows PCA analysis of 37 saliva samples and it can be observed that PCA enabled the distribution of samples based on their presence or absence of PD. Furthermore, it was observed that SPGE grouped the saliva samples according to their condition, meaning that the detected currents vary significantly based on the levels of MPO present in the samples. This suggests a potential link between MPO levels and PD, highlighting the utility of SPGE in detecting and monitoring biomarkers associated with oral health.

## Discussion

Figure 2 demonstrates a linear relationship between the square root of the scan rate and both the anodic and cathodic peak currents, indicating that the electroactive species undergo a surface-controlled quasi-reversible process as they move toward the electrode surface [33]. This behavior has been observed in other studies based on SPGE modified with peroxidases from different sources [14]. The CV experiments (Fig. 2A) revealed a notable increase in current at the cathodic peak with higher concentrations of peroxide applied to the electrodes, each coated with the same concentration of MPO. This observation suggests a direct correlation between peroxide concentration and the electrochemical response mediated by MPO. Importantly, this behavior signifies the absence of inhibitory effects, indicating that a wide range of peroxide concentrations can be effectively utilized in such systems without compromising enzymatic activity [38]. In addition, comparative analysis of the CVs obtained for different peroxide concentrations allowed for the selection of an optimal concentration of 0.5 mM (Fig. 2A, green color), based on its symmetry. This optimal profile ensures a more controlled and reproducible electrochemical response, minimizing potential distortions in the obtained data. Once the substrate concentration was selected, its behavior in the presence and absence of the MPO was studied. This characterization is depicted in Fig. 3B, where an increase in cathodic current is observed once the MPO is attached to the surface of the electrodes. This increase is perceived as a favorable response, representing the catalytic action of MPO in the presence of H_2_O_2_. The CV experiments from Fig. 3A revealed a notable increase in current at the cathodic peak with higher concentrations of peroxide applied to the electrodes, each coated with the same concentration of MPO. This observation suggests a direct correlation between peroxide concentration and the electrochemical response mediated by MPO. Importantly, this behavior signifies the absence of inhibitory effects, indicating that a wide range of peroxide concentrations can be effectively utilized in such systems without compromising enzymatic activity [38]. In addition, comparative analysis of the CVs obtained for different peroxide concentrations allowed for the selection of an optimal concentration of 0.5 mM (green color), based on its symmetry. This optimal profile ensures a more controlled and reproducible electrochemical response, minimizing potential distortions in the obtained data. Once the substrate concentration was selected, its behavior in the presence and absence of the MPO was studied. This characterization is depicted in Fig. 3B, where an increase in cathodic current is observed once the MPO is attached to the surface of the electrodes. This increase is perceived as a favorable response, representing the catalytic action of MPO in the presence of H_2_O_2_.

The pivotal role of H_2_O_2_ as a mediator for catalytic activity on the electrode surface [39] underscores the potential utility of the proposed biosensing platform for detecting MPO levels in saliva samples, even in the absence of a definitive PD diagnosis. Furthermore, it was observed that SPGE grouped the saliva samples according to their condition (Fig. 6), meaning that the detected currents vary significantly based on the levels of MPO present in the samples. This suggests a potential link between MPO levels and PD, highlighting the utility of SPGE in detecting and monitoring biomarkers associated with oral health.

## Conclusion

In conclusion, this study underscores the potential of MPO as a robust biomarker for PD and the utility of electrochemical sensing coupled with principal component analysis (PCA) for detection in clinical samples. By employing SPGE, we have demonstrated a novel approach to detect MPO, offering a promising avenue for early diagnosis and monitoring of PD. Our results not only enhance our understanding of the electrochemical properties of MPO but also lay the groundwork for creating effective diagnostic tools for oral health issues. With further refinement and validation, this methodology holds significant promise for improving patient outcomes through timely intervention and management of periodontal disease.

Despite the promising results demonstrated in this study, several limitations should be acknowledged. First, while the electrochemical sensor demonstrated effective detection of MPO in saliva samples, the sensitivity and selectivity of the sensor may be influenced by the complex nature of biological fluids. The potential interference from other components in saliva, such as proteins and other biomarkers, was not fully explored and could affect the accuracy of the MPO measurements. Future studies should investigate strategies for minimizing such interferences, including further optimization of the electrode surface or the use of selective binding agents.

## Data Availability

The data supporting the findings of this study are available from the corresponding author upon reasonable request. All datasets have been anonymized to ensure patient confidentiality and comply with ethical guidelines.
